# Supplementary material for: Automatic real-time analysis and interpretation of arterial blood gas sample for Point-of-care testing: Clinical validation
Source: PLoS One. 2021 Mar 10;16(3):e0248264. doi: 10.1371/journal.pone.0248264 (PMC7946183; doi:10.1371/journal.pone.0248264)
Supplement: S1 Table — (DOCX) [file pone.0248264.s001.docx]

**S1 Table.**

**NORMAL**

0 'Normal acid-base status'

**RESPIRATORY ALKALOSIS**

1. 'Alkalemia; acute respiratory alkalosis; simple disorder'

2. 'Alkalemia; chronic respiratory alkalosis; simple disorder'

3. 'Alkalemia; acute respiratory alkalosis; complex disorder; concomitant metabolic alkalosis'

4. 'Alkalemia; chronic respiratory alkalosis; complex disorder; concomitant metabolic alkalosis'

5. 'Alkalemia; acute respiratory alkalosis; complex disorder; concomitant normal AG metabolic acidosis'

6.'Alkalemia; acute respiratory alkalosis; complex disorder; concomitant abnormal AG metabolic acidosis'

7.'Alkalemia; acute respiratory alkalosis; complex disorder; concomitant normal and abnormal AG metabolic acidosis'

8.'Alkalemia; acute respiratory alkalosis; complex disorder; concomitant abnormal AG metabolic acidosis and metabolic alkalosis'

9.'Alkalemia; chronic respiratory alkalosis; complex disorder; concomitant normal AG metabolic acidosis'

10.'Alkalemia; chronic respiratory alkalosis; complex disorder; concomitant abnormal AG metabolic acidosis'

11.'Alkalemia; chronic respiratory alkalosis; complex disorder; concomitant normal and abnormal AG metabolic acidosis'

12.'Alkalemia; chronic respiratory alkalosis; complex disorder; concomitant abnormal AG metabolic acidosis and metabolic alkalosis'

**METABOLIC ALKALOSIS**

13. 'Alkalemia; metabolic alkalosis; simple disorder'

14. 'Alkalemia; metabolic alkalosis; complex disorder; concomitant respiratory acidosis'

15. 'Alkalemia; metabolic alkalosis; complex disorder; concomitant respiratory alkalosis'

**METABOLIC ACIDOSIS**

16. 'Acidemia; normal AG metabolic acidosis; simple disorder'

17. 'Acidemia; abnormal AG metabolic acidosis; simple disorder'

18. 'Acidemia; normal and abnormal AG metabolic acidosis; simple disorder'

19. 'Acidemia; normal AG metabolic acidosis; complex disorder; concomitant respiratory acidosis'

20. 'Acidemia; abnormal AG metabolic acidosis; complex disorder; concomitant respiratory acidosis'

21. 'Acidemia; normal and abnormal AG metabolic acidosis; complex disorder; concomitant respiratory acidosis'

22. 'Acidemia; normal AG metabolic acidosis; complex disorder; concomitant respiratory alkalosis'

23. 'Acidemia; abnormal AG metabolic acidosis; complex disorder; concomitant respiratory alkalosis'

24. 'Acidemia; normal and abnormal AG metabolic acidosis; complex disorder; concomitant respiratory alkalosis'

**RESPIRATORY ACIDOSIS**

25. 'Acidemia; acute respiratory acidosis; simple disorder'

26. 'Acidemia; chronic respiratory acidosis; simple disorder'

27. 'Acidemia; acute respiratory acidosis; complex disorder; concomitant metabolic alkalosis'

28. 'Acidemia; chronic respiratory acidosis; complex disorder; concomitant metabolic alkalosis'

29. 'Acidemia; acute respiratory acidosis; complex disorder; concomitant normal AG metabolic acidosis'

30. 'Acidemia; acute respiratory acidosis; complex disorder; concomitant abnormal AG metabolic acidosis'

31. 'Acidemia; acute respiratory acidosis; complex disorder; concomitant normal and abnormal AG metabolic acidosis'

32. 'Acidemia; acute respiratory acidosis; complex disorder; concomitant abnormal AG metabolic acidosis and metabolic alkalosis'

33. 'Acidemia; chronic respiratory acidosis; complex disorder; concomitant normal AG metabolic acidosis'

34. 'Acidemia; chronic respiratory acidosis; complex disorder; concomitant abnormal AG metabolic acidosis'

35. 'Acidemia; chronic respiratory acidosis; complex disorder; concomitant normal and abnormal AG metabolic acidosis'

36. 'Acidemia; chronic respiratory acidosis; complex disorder; concomitant abnormal AG metabolic acidosis and metabolic alkalosis'
