# Supplementary material for: Automatic real-time analysis and interpretation of arterial blood gas sample for Point-of-care testing: Clinical validation
Source: PLoS One. 2021 Mar 10;16(3):e0248264. doi: 10.1371/journal.pone.0248264 (PMC7946183; doi:10.1371/journal.pone.0248264)
Supplement: S1 Text — (DOCX) [file pone.0248264.s002.docx]

**S2. Text. Three clinical cases to ilustrate the ABG-a use.**

**Three Case Examples**

**Case 1**

A 52-year-old Afro-Caribbean man with a past medical history of Sleep Apnoea Syndrome (SAS) and overweight presented to the Emergency Department (ED) with a history of being unwell for the last three days, vomiting, unable to eat and drink, with a high temperature of 39°C (102,2 °F). On admission, he was clammy and had a remarkable work of breathing. His initial observations were recorded as heart rate (HR) 115/min; blood pressure (B/P) 130/65; urine output (UO) 20 ml in the last hour post insertion of the urinary catheter in the ED. His chest X-ray showed bilateral infiltrates. A full set of bloods (including urea and creatinine, full blood count and clotting), an arterial blood gas (ABG) and a PCR for COVID-19 were simultaneously taken. He was administered oxygen via a non-rebreathe mask with FiO_2_ 100% giving oxygen saturations of 86% and showing no signs of improvement in his work of breathing or oxygenation. While the formal blood results were being processed in the laboratory the instant results of the ABG were available; Sodium 149mmol/L (149mEq/L), Potassium 3.5mmol/L (3.5mEq/L), Chloride 105mmol/L (105mEq/L), Lactate of 3mmol/L (3mEq/L), Glucose 4.2mmol/L (75.67mg/dL), Bicarbonate 25.9mmol/L (25.9mEq/L) and SBE -3. The arterial blood pH was 7.20, PaCO_2_ 65.62mHg (8.75 kPa) and PO_2_ 77.25mmHg (10.3kPa). The renal function from the ABS was Urea 25mmol/L (150.17mg/dL), Creatinine 144**µ**mol/L (1.63mg/dL). The ABG-a (Arterial Blood Gas Algorithm) used at the POC testing of the ABG within the ED reported a preliminary and differential diagnosis (Fig 1 and Fig 2).


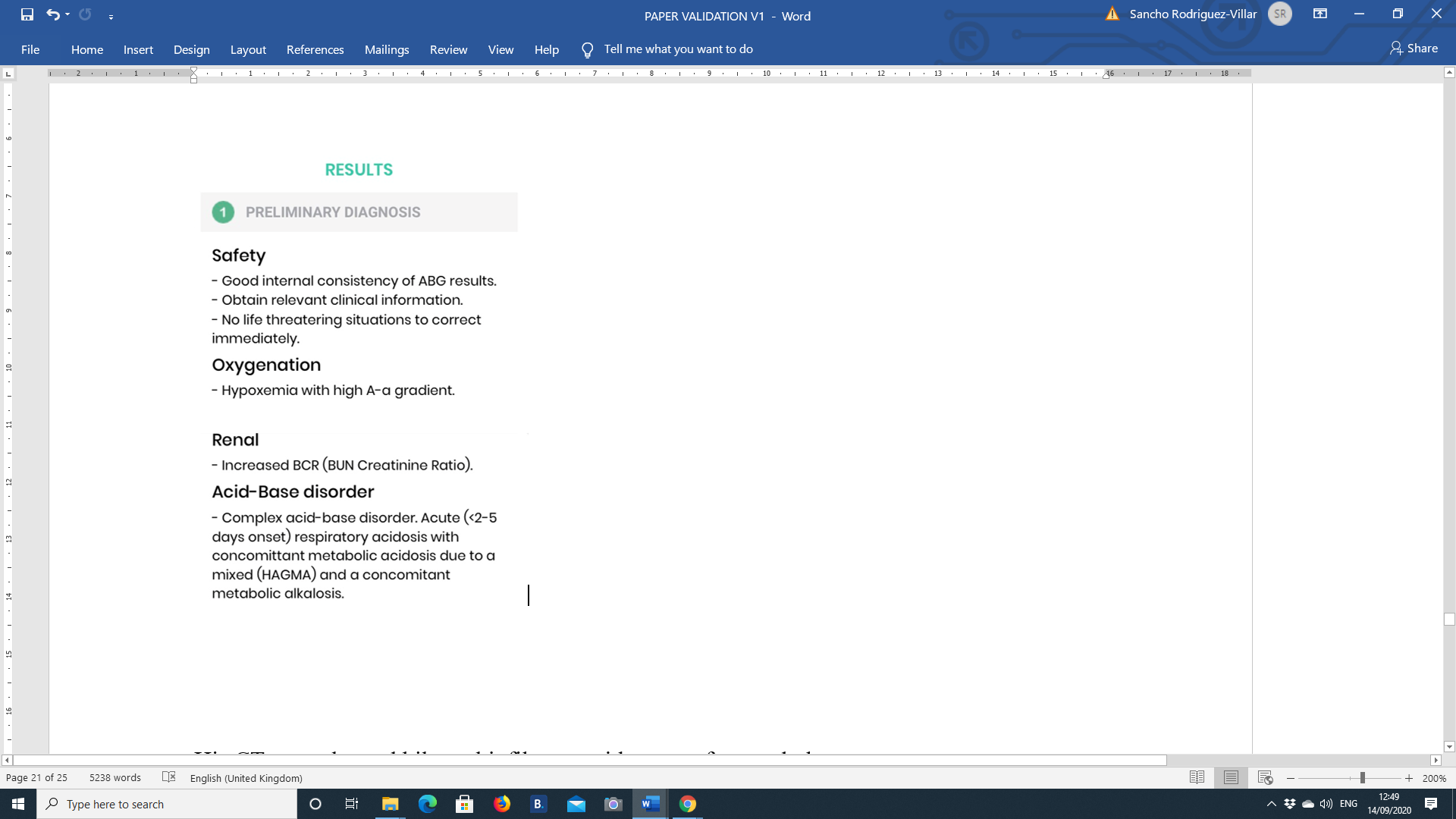


Fig 1: Preliminary Diagnosis from ABG-a.


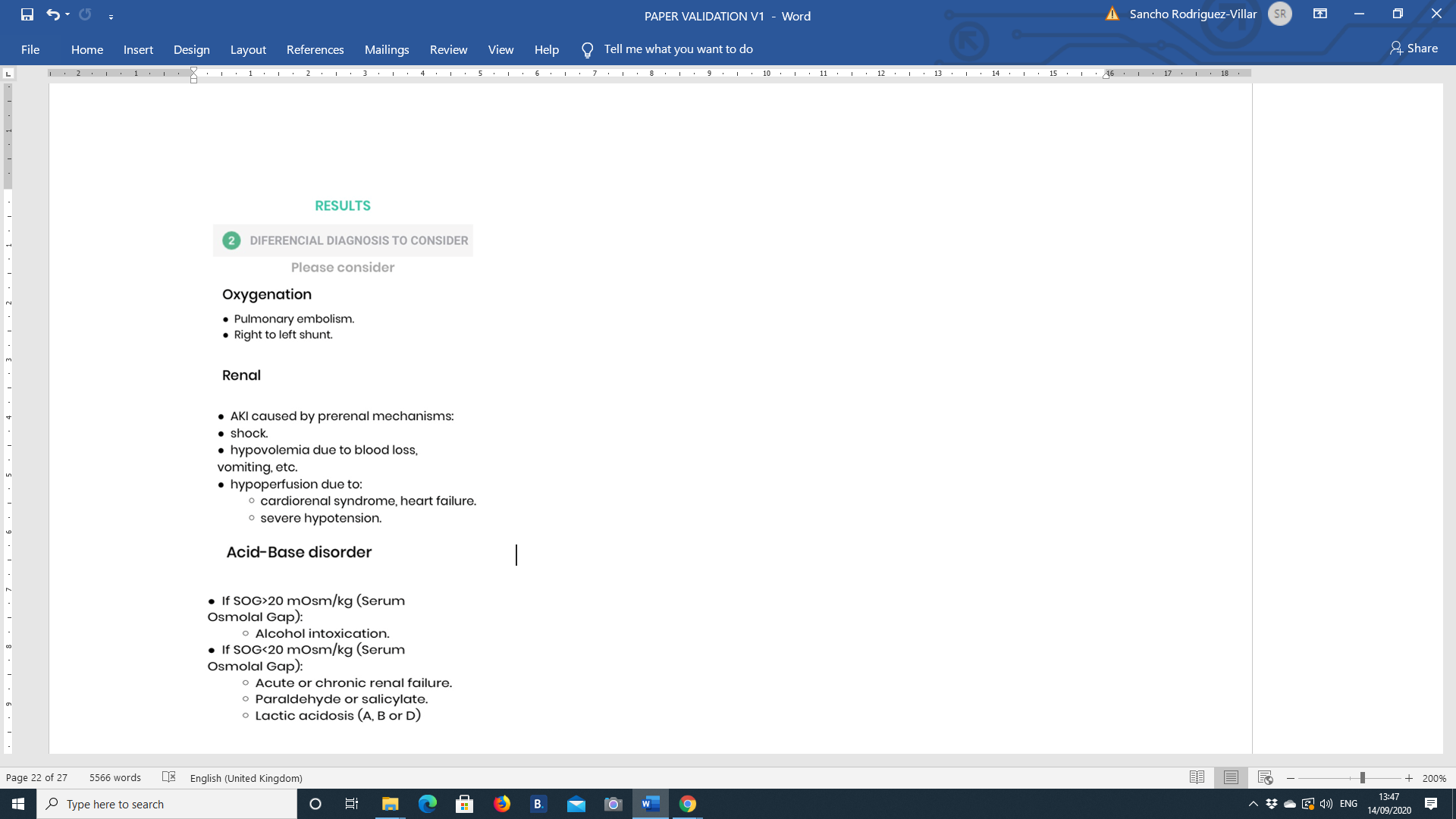


Fig 2: Differential Diagnosis from ABG-a.

The preliminary diagnosis report (Fig 1) is presented in four sections: safety, oxygenation, renal and acid-base disorder. The report firstly focuses on safety by showing the clinician that the results can be trusted since they are reported with a ‘good internal consistency’. This means the results fit a clinical picture and do not show a number of erroneous results, which would immediately question the validity of the sample. The next step in the safety report suggests the clinician correlates the results with the current clinical presentation. The final section of the safety reports indicates that there are no imminent life-threatening indications that require immediate management from the blood sample processed. The next section, oxygenation, demonstrates the patient has hypoxemia with a high alveolar-arterial gradient fitting with the clinical picture and oxygen administration requirements. The renal analysis shows an increased BCR (Bun Creatinine Ratio) meaning the urea level is disproportionally higher than the increased plasma creatinine. This again fits the clinical presentation of high fever and severe dehydration from not being able to drink for the last few days. The final section of the report focuses on the acid-base disorder. In this case, a complex acid-base disorder is shown from a respiratory acidosis with an associated high anion gap and therefore metabolic acidosis alongside an associated metabolic alkalosis. As discussed previously, despite the correct description from the ABG-a, due to the overlap of clinical events, time-lag and multiorgan involvement in this clinical case, it is almost impossible to tell with absolute certainly which is the primary or secondary acid-base disorder despite we agree with the ABG-a. However, the ABG-a fulfil the aim which is to provide us with a detailed interpretation of the disturbances involved.

The differential diagnosis report (Fig 2) is presented in three sections providing the most likely causes, in relation to oxygenation, renal and acid-base disorder. At this stage these likely causes are based on the initial ABG and could be used to guide the clinician in evaluating the suggestions provided by the ABG-a alongside the clinical presentation.

The differential diagnosis suggests a pulmonary embolism (PE) with a pulmonary shunt, acute kidney injury (AKI) of a pre-renal cause due to hypovolaemia and likely as a result of the dehydration due to a lack of oral intake and fever and an acid-base disorder to include lactic acidosis and renal failure. This would suggest the need for a CT scan with contrast to determine the diagnosis of a PE and a plasma osmolality to determine the metabolic cause and therefore appropriate treatment.

In this case study the CT-scan findings showed bilateral pulmonary embolisms, bilateral infiltrates with areas of ground glass and was treated with a heparin infusion.

**Case 2**

A 35-year-old woman who had been involved in a motorbike accident, arrived to ED (Emergency Department). She was hypoperfused peripherally with initial observations recorded as: HR 122 bpm, B/P 90/55, UO 30 ml in the last hour after the urinary catheter was inserted in ED on arrival.

She received 8 litres of isotonic saline for resuscitation. A full set of bloods (including urea and creatinine, full blood count, clotting and cross match) and an arterial blood sample (ABS) were taken simultaneously. The results of the ABS are available almost immediately due to point-of-care testing however the full set of bloods take slightly longer due to being processed in the hospital laboratory. The ABG showed the following: Sodium 135mmol/L, Potassium 3.8mmol/L, Chloride 115mmol/L, Lactate 1mmol/L (1mEq/L) and Bicarbonate 18mmol/L. The arterial blood pH was 7.28, PaCO_2_ 39mmHg (5.2kPa) and the PO_2_ 67.5mmHg (9kPa) on room air. The renal function from the ABS showed Urea 15mmol/L (42.01mg/dL), Creatinine 150 **µ**mol/L (1.69mg/dL). The ABG-a produces two reports on the preliminary and differential diagnosis (Fig 3 and Fig 4) and is available immediately post ABG processing as part of ABG point-of-care testing.


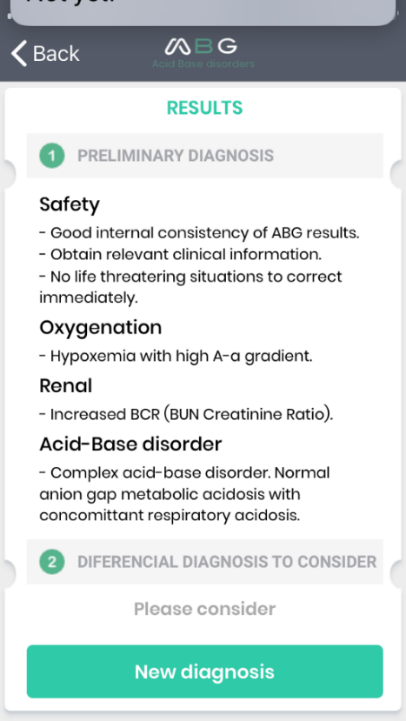


Fig 3: Preliminary Diagnosis from ABG-a.


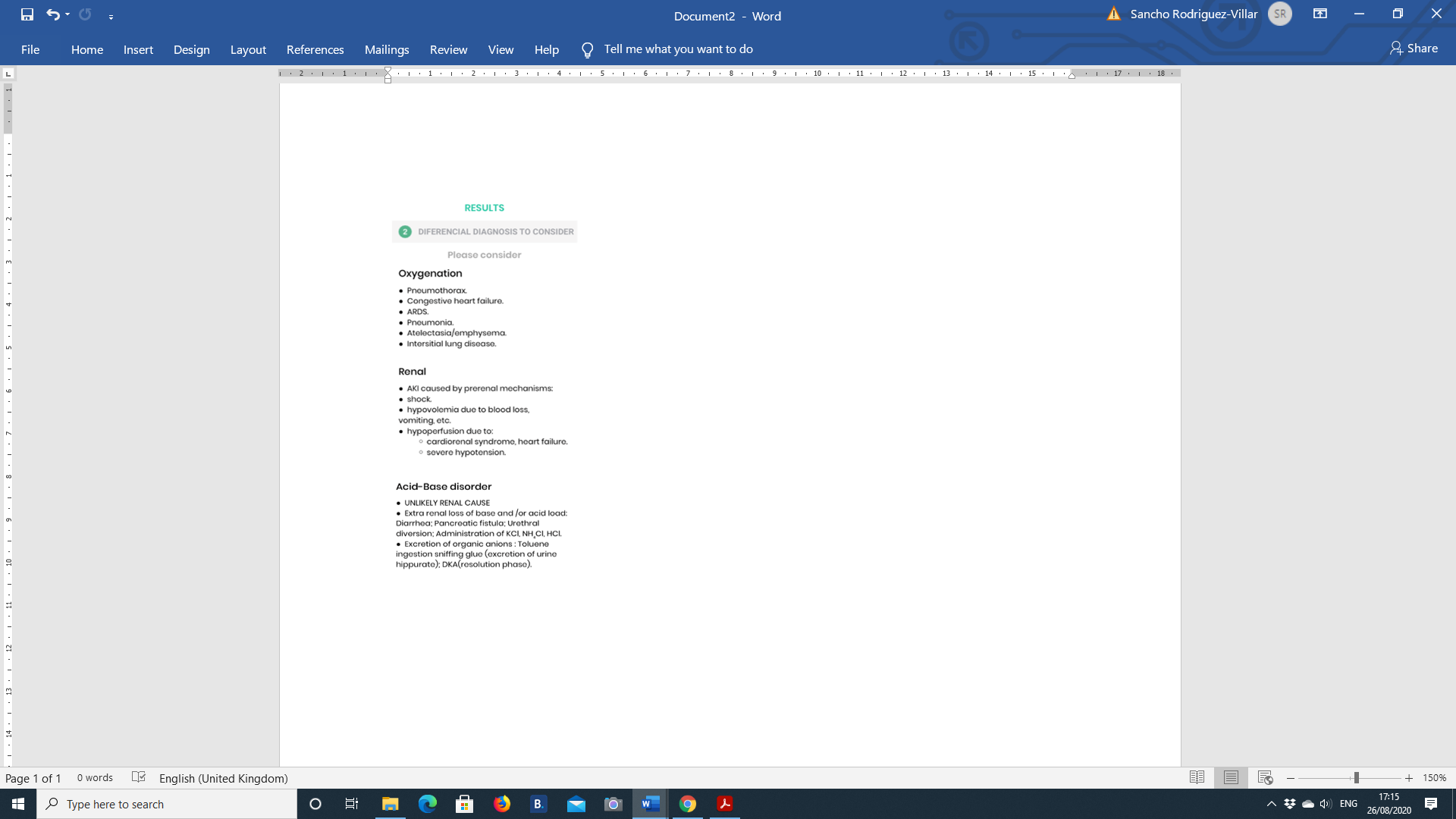


Fig 4: Differential Diagnosis from ABG-a.

The preliminary diagnosis report (Fig 3) is presented in four sections: safety, oxygenation, renal and acid-base disorder. The report firstly focuses on safety by showing the clinician that the results can be trusted since they are reported with a ‘good internal consistency’. This means the results fit a clinical picture and do not show a number of erroneous results, which would immediately question the validity of the sample. The next step in the safety report suggests the clinician correlates the results with the current clinical presentation. The final section of the safety reports indicates that there are no imminent life-threatening indications that require immediate management from the blood sample processed.

The preliminary report (Fig 3) suggests the patient had a respiratory acidosis with an increased BCR (Bun Creatinine Ratio) and a normal anion-gap metabolic acidosis. This fits with the clinical presentation of blood loss and shock.

The differential diagnosis report (Fig 4) is presented in three sections providing the most likely causes, in relation to oxygenation, renal and acid-base disorder. At this stage these likely causes are based on the initial ABG and should be used to guide the clinician in evaluating the suggestions provided by the ABG-a alongside the clinical presentation.

The differential diagnosis report in Fig 4 suggests a pneumothorax. This was the finding in the case due to three rib fractures resulting in a pneumothorax. The report suggests an AKI of pre-renal cause and is likely secondary to shock due to hypovolaemia. In practice, the majority of patients with a normal anion-gap metabolic acidosis have diarrhoea and renal tubular acidosis, however, in this case that does not fit the presentation and the clinician must consider the clinical context.

As the team were preparing to insert a chest drain results from the biochemistry lab were reported. The urinary Sodium level was 65mmol/L, Potassium 15mmol/L, Urine Osmolality 1500mOsm/kg and Chloride 110mmol/L, Osmolality 1500mOsm/L and albumin 28g/L. These results were added to the software and a new differential diagnosis report was issued (Fig 5).


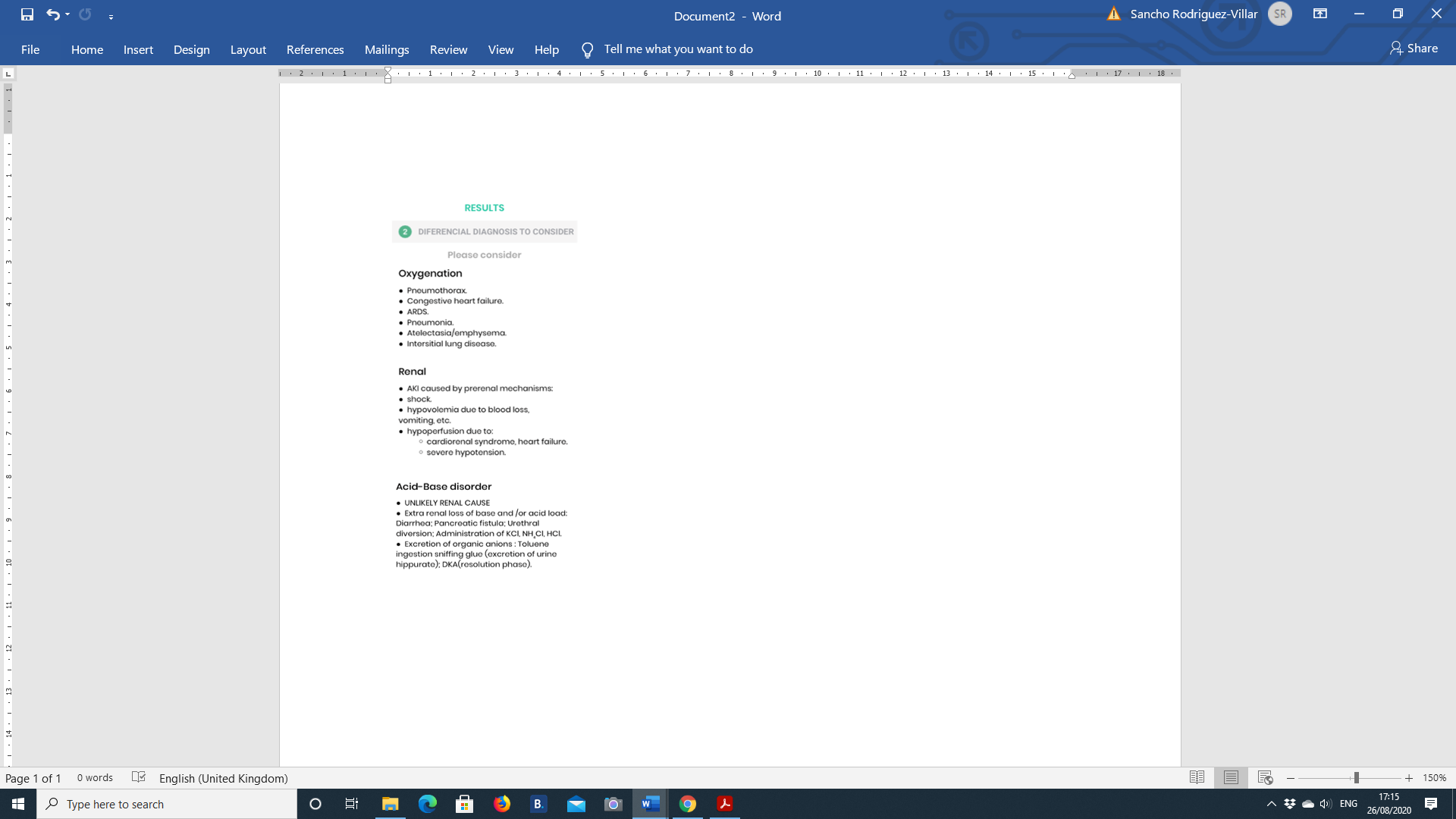


Fig 5: Final Differential Diagnosis Report from ABG-a.

The high chloride content of the normal saline induces a non-anion gap metabolic acidosis (NAGMA), decreasing the bicarbonate concentration. Finally, the low AG is probably the result of a low albumin level because of the acute bleeding and dilution. The updated reported therefore ruled out an intrinsic renal cause and suggested that the aetiology of the disturbance was the administration of a Cl^-^ rich solution.

**Case 3**

A 62-year-old woman discharged from ICU developed in the last 24 hours, large volumes of watery diarrhoea during her stay on the medical ward and tested positive to Clostridium difficile. She was clinically dehydrated with initial observations recorded as: HR 105 bpm, B/P 115/65 and temperature of 38.3°C (100.4 °F).

The ABG showed the following: Sodium 140mmol/L, Potassium 3mmol/L, Chloride 86mmol/L, Lactate 1mmol/L (1mEq/L) and Bicarbonate 38mmol/L. The arterial blood pH was 7.61, PaCO_2_ 40mmHg (5.3kPa) and the PO_2_ 99.67mmHg (13.2kPa) on room air. The renal function from the ABG showed Urea 15mmol/L (42.01mg/dL), Creatinine 150**µ**mol/L (1.69mg/dL). The ABG-a produced two reports on the preliminary and differential diagnosis (Fig 6 and Fig 7).


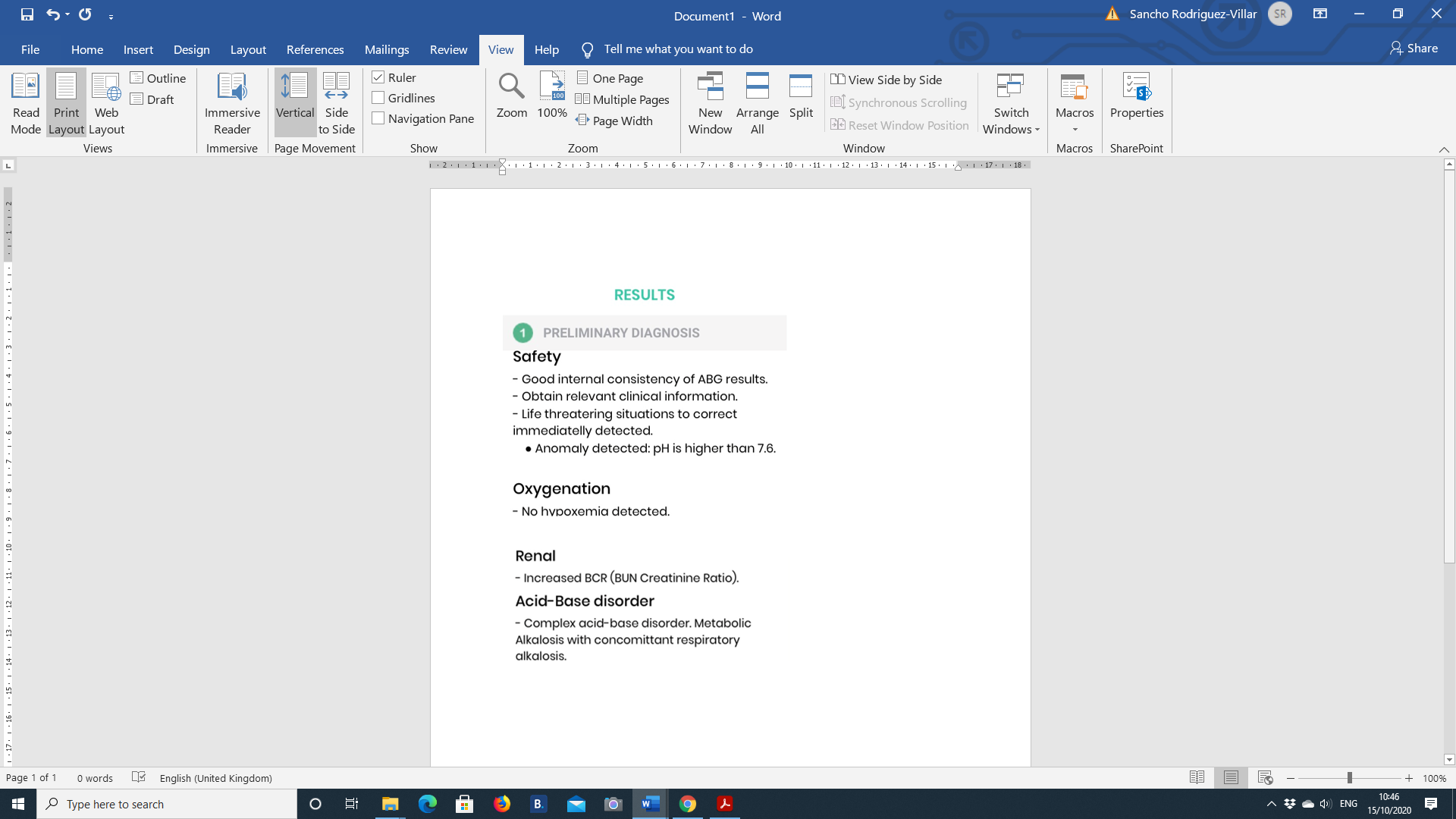


Fig 6. Preliminary Diagnosis from ABG-a.


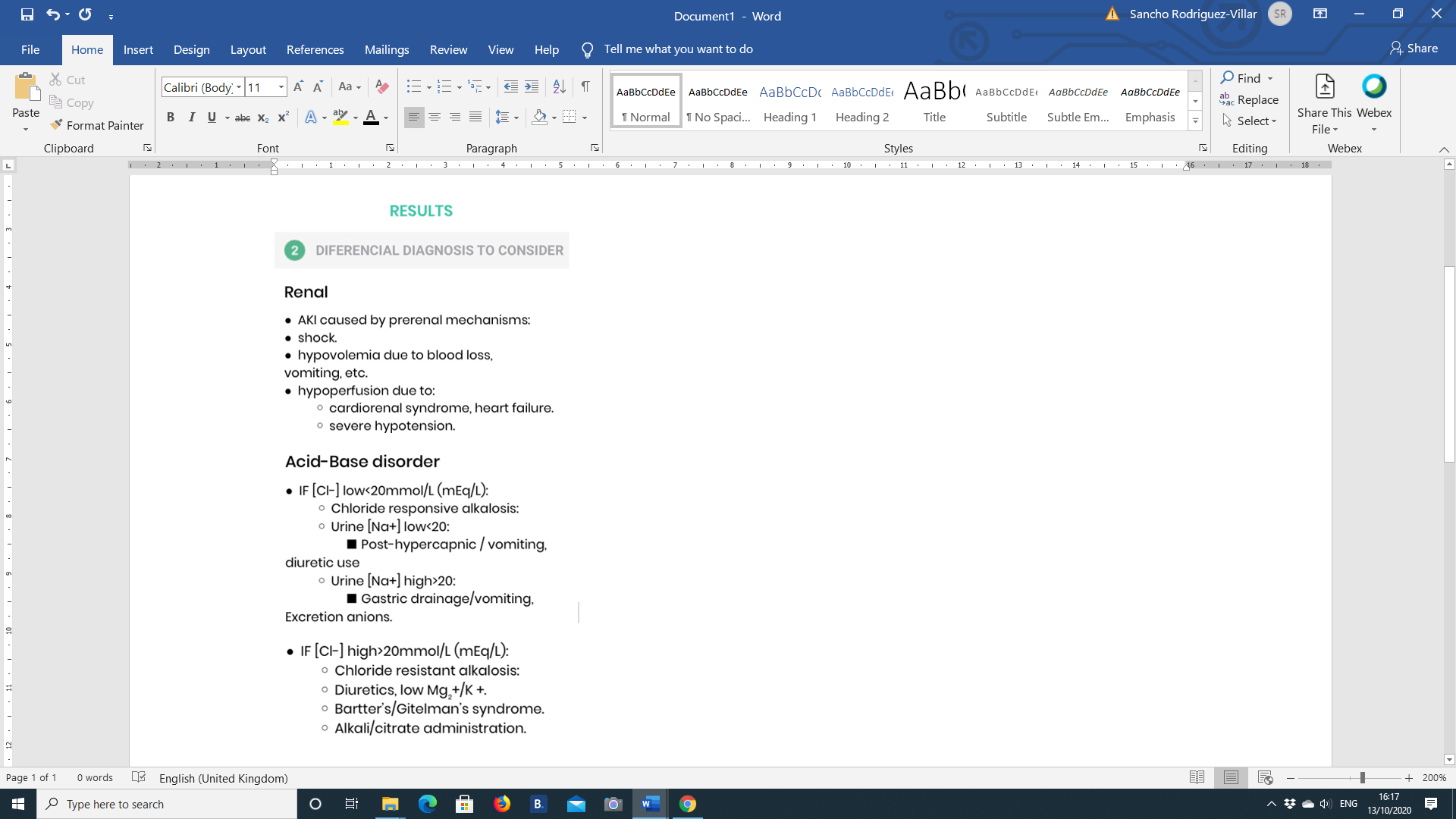


Fig 7. Differential Diagnosis from ABG-a.

The ABG-a highlights a life-threatening situation (pH>7.61) in the safety section. She has a metabolic alkalosis as a result of the gastrointestinal losses but also has a respiratory alkalosis, perhaps due to hyperventilation secondary to fever, pain and stress. The ABG-a also suggests to check for electrolytes (Urine Cl^-^) in order to confirm the diagnosis in the clinical context.
