# Supplementary material for: Automatic real-time analysis and interpretation of arterial blood gas sample for Point-of-care testing: Clinical validation
Source: PLoS One. 2021 Mar 10;16(3):e0248264. doi: 10.1371/journal.pone.0248264 (PMC7946183; doi:10.1371/journal.pone.0248264)
Supplement: Attachment — Submitted filename: Response to Reviewers 09_02_21.docx [file pone.0248264.s005.docx]

06/02/21

REBUTTAL LETTER

Dear Academic Editor and Reviewers,

**RE: PONE-D-20-32648**

**Automatic real-time analysis and interpretation of arterial blood gas for Point-of-Care testing: clinical validation**

Thank you for reviewing and providing feedback to the paper. We will try to answer all your comments in a rigorous fashion by order.

6. Review Comments to the Author

Please use the space provided to explain your answers to the questions above. You may also include additional comments for the author, including concerns about dual publication, research ethics, or publication ethics. (Please upload your review as an attachment if it exceeds 20,000 characters)

Reviewer #1: (No Response)

Reviewer #3: I think this paper is very interesting and well written. But, I have only one serious concern.

According to Tables 7 and 8, both median and IQR are in the normal range. What are the values of the raw data that is the basis of this analysis? The author should show raw blood gas data as shown in PLOS Data policy.

Anonymous raw blood gas data showed as per PLOS Data policy. Please, see attached .

Best regards

Dr Sancho Rodriguez-Villar

12/12/20

REBUTTAL LETTER

Dear Academic Editor and Reviewers,

**RE: PONE-D-20-32648
Automatic real-time analysis and interpretation of arterial blood gas for Point-of-Care testing: clinical validation**

Thank you for reviewing and providing feedback to the paper. We will try to answer all your comments in a rigorous fashion by order.

**Reviewer #1: Based on the results, the arterial blood gas algorithm (ABG-a) is practical for acute and critical care. Only one question are needed for explanation and one suggestion are recommended.

*Question:* Please explain the relatively low sensitivity in respiratory acidosis (61.1%), metabolic acidosis (75.8%), and metabolic alkalosis (72.2%).**

From the eight acid-base disorders, only three of them have a sensitivity of less than 80%. In the diagnosis of acid-base disorders, there is really no diagnostic test considered as reference “Gold standard” except for an expert opinion.

Firstly, it is very remarkable that only this relatively low sensitivity occurs with those acid-base disorders classified as “single” and not “complex” acid-base disorders. In our opinion based on the observations during the study, we can appreciate that the software is able to run calculations on extremely mild deranged values from the arterial blood gases, and therefore can obtain more refined conclusions than that of the clinicians. Also, the ABG-a follows the rules of certain values cutting points. The clinicians however only consider the presence of “complex” acid-base disturbances when the results are very obviously deranged. This approach from the clinicians maybe explained by many reasons, not least of all limited time for analysing every single ABG result in such a rigorous and complex manner. The diagnostic judgment of experienced clinicians has been taken as a reference test, which without denying its validity, can change from one clinician to another. Further studies in this regard would be necessary, after which it would be possible to consider our algorithm as a reference test.

Secondly, those diagnosis made by the ABG-a as “complex” still include the primary diagnosis of “respiratory acidosis”, “metabolic acidosis” and “metabolic alkalosis”. Not only keeping the safety standards but also improving the diagnosis. We know that in the human physiology, the presence of disturbances are complex and frequently not simple, making the ABG-a of great value in order to help with a more refined diagnosis.

***Suggestion:* The data (median & IQR) from enrolled samples in Table 7 & 8 seem so normal. The reports of minimum and maximum levels of tests may be demonstrated, as a kind of description of the severity and variability of tested samples.**

We could understand that the suggestion relates to utilising means and standard deviations as opposed to medians and interquartile ranges because this form still present in some publications, but asking for the maximum and minimum values to describe and represent a quantitative variable is in our opinion inappropriate. The current trend is to describe quantitative values with median and interquartile range because they do not require compliance with normality; they are very robust, represent the data well and are not affected by the presence of extreme values. On the contrary, if they were described with the range and the minimum and maximum values, the representativeness of the data would be violated since those minimum and maximum values are usually atypical and remote values.

***Some errors need to correct:*
Table 4: Acute renal failure or acute on chronic renal failure ◊ acute kidney injury or acute on chronic kidney disease
Table 8: 4,1 --> 4.1
Line 352: 83,4%--> 83.4%**

We agree and these errors have been corrected on the manuscript.

**Reviewer #2:**

**Additionally, reporting the global accuracy in this type of work is misleading. The dataset is unbalanced (many more negative cases than positive cases, for at least some of the choices of acid base disorder. Without knowledge of the actual numbers of diagnoses (how many negative, how many metabolic acidosis, etc) it is impossible to interpret global accuracy, and it is difficult to assess the importance of poor performance on some disease states. I would request that the authors include the number of patients with each disorder; this would be part of the PLOS requirement that "all data be made available". Additionally, reporting a beta-weighted F-score may give a more interpretable level of global accuracy, with beta chosen to reflect the relative cost between a false negative and a false positive.**

We agree that reporting global accuracy can be misleading and that reporting the frequency of each of the acid-base disorders helps with interpretation of the data. Table 10 has been modified to include a column with the number of cases of each of the acid-base disorders. It is observed that since there are fewer positive than negative cases, the sensitivity values are lower than those of specificity and the 95% CIs are wider in sensitivity and narrower in specificity. By reporting the global accuracy together with the sensitivity and specificity as well as the frequency of each disorder, as reflected in the modified table 10, there is less misunderstanding. The beta-weighted F-score could have been used as suggested, although it has the drawbacks that it ignores true negatives in its calculation and assigns equal importance to precision and recall. In our work we have chosen the analysis of reliability with the kappa index and accuracy with sensitivity, specificity and, together with them, and not in isolation, the global accuracy.

**PLOS authors have the option to publish the peer review history of their article (**[**what does this mean?**](https://journals.plos.org/plosone/s/editorial-and-peer-review-process#loc-peer-review-history)**). If published, this will include your full peer review and any attached files.**

We are agreed as authors to be published our article peer review history

Best regards

Dr Sancho Rodriguez-Villar
